# Microglial function, INPP5D/SHIP1 signaling, and NLRP3 inflammasome activation: implications for Alzheimer’s disease

**DOI:** 10.1186/s13024-023-00674-9

**Published:** 2023-11-29

**Authors:** Gizem Terzioglu, Tracy L. Young-Pearse

**Affiliations:** Ann Romney Center for Neurologic Diseases, Department of Neurology, Brigham and Women’s Hospital and Harvard Medical School, 60 Fenwood Rd, Boston, MA 02115 USA

**Keywords:** NLRP3 inflammasome, SHIP1, INPP5D, Alzheimer’s Disease, Inflammation, Phosphoinositide signaling, Microglia

## Abstract

Recent genetic studies on Alzheimer’s disease (AD) have brought microglia under the spotlight, as loci associated with AD risk are enriched in genes expressed in microglia. Several of these genes have been recognized for their central roles in microglial functions. Increasing evidence suggests that SHIP1, the protein encoded by the AD-associated gene *INPP5D*, is an important regulator of microglial phagocytosis and immune response. A recent study from our group identified SHIP1 as a negative regulator of the NLRP3 inflammasome in human iPSC-derived microglial cells (iMGs). In addition, we found evidence for a connection between SHIP1 activity and inflammasome activation in the AD brain. The NLRP3 inflammasome is a multiprotein complex that induces the secretion of pro-inflammatory cytokines as part of innate immune responses against pathogens and endogenous damage signals. Previously published studies have suggested that the NLRP3 inflammasome is activated in AD and contributes to AD-related pathology. Here, we provide an overview of the current understanding of the microglial NLRP3 inflammasome in the context of AD-related inflammation. We then review the known intracellular functions of SHIP1, including its role in phosphoinositide signaling, interactions with microglial phagocytic receptors such as TREM2 and evidence for its intersection with NLRP3 inflammasome signaling. Through rigorous examination of the intricate connections between microglial signaling pathways across several experimental systems and postmortem analyses, the field will be better equipped to tailor newly emerging therapeutic strategies targeting microglia in neurodegenerative diseases.

## Background

Alzheimer’s disease (AD) is a neurodegenerative disease that accounts for an estimated 60–80% of dementia cases in the United States. Alzheimer’s dementia presents with memory loss, apathy, confusion, impaired communication and behavioral changes. Pathologically, AD is characterized by the presence of extracellular amyloid β (Aβ) plaques, intracellular neurofibrillary tau tangles, neuroinflammation, synapse loss and cell death. Most AD cases arise due to an interplay between complex polygenic mechanisms and non-genetic factors [1, 2]. In recent years, genome wide association studies (GWASs) and analyses of rare-coding variants have identified over 70 loci associated with conferring risk for late-onset AD (LOAD). Many of these loci are in the vicinity of genes that are strongly expressed in microglia and implicated in microglial functions, including *ABCA7, ABI3, APOE, CD2AP, CD33, CLU, CR1, EPHA1, HLA-DRB5/DBR1, MS4A6A/MS4A6E, PLCG2, SORL1, TREM2* and *INPP5D* [3–11]. An active area of investigation is geared towards unraveling the precise mechanisms by which these variants increase risk for AD. Elucidating the normal function of each of the genes associated with these loci will enlighten our understanding of microglia’s role in the adult brain. Here we focus primarily on what is known about one of these genes, *INPP5D*, in microglial function and dysfunction.

Microglia are the resident myeloid cells of the brain parenchyma. They survey and sense their local environment using their dynamic, fine processes and respond to stimuli including neuronal and other cellular activity, cell death, injury, infection and other inflammatory insults [12, 13]. During development, microglia are involved in shaping neuronal circuits by pruning and remodeling synapses, clearing dead or dying cells, and regulating myelination and vasculature development by interacting with other glial and endothelial cells of the central nervous system (CNS) [12, 14, 15]. In adulthood, microglia exist as a heterogenous population of cells that can display distinct phenotypes and functions. While some microglia continue to exhibit a homeostatic phenotype to maintain brain homeostasis, some may change dramatically as they respond to injury or insults by releasing pro-inflammatory cytokines, causing neurotoxicity directly or inducing neurotoxic phenotypes in astrocytes [16–18]. While “healthy” microglia play important roles in protecting the brain, in neurodegenerative disease, “dysfunctional” microglia can contribute to further pathology. An array of cell surface receptors and signaling pathways in microglia have been implicated in mechanisms underlying AD. While the literature can be confusing at times with seemingly conflicting data regarding the precise mechanisms at play in microglia, examination of the data leads to two high level conclusions: (1) there is complex regulation of microglial activation that involves both negative and positive feedback and (2) cellular and molecular context plays a critical role in determining the precise pathway(s) affected in microglia in AD. In this review, we attempt to integrate findings regarding *INPP5D* with the growing literature regarding microglial pathways that impinge upon risk for AD.

One mechanism by which multiple cell types contribute to inflammatory processes that is increasingly gaining attention is the activation of the nucleotide-binding domain (NOD)-like receptor protein 3 (NLRP3) inflammasome. Inflammasomes are intracellular molecular platforms that induce the secretion of pro-inflammatory cytokines to engage the innate immune system against infection or cellular stress [19]. In this review, we describe the formation and functions of the microglial NLRP3 inflammasome, particularly in the context of neuroinflammatory processes in AD. We then summarize the known functions of a gene expressed exclusively in myeloid cells that is associated with LOAD through GWAS, *INPP5D*. We discuss the role of SHIP1, the protein encoded by *INPP5D*, in phosphoinositide signaling and its involvement in microglial functions, and recent evidence suggesting that reduction of SHIP1 activity results in activation of the NLRP3 inflammasome. Finally, we hypothesize a mechanistic link between SHIP1’s regulation of the NLRP3 inflammasome and microglial function, and the implications of this regulation on different cell types in the brain.

## Role of inflammation in AD

Aging, which remains the largest risk factor for LOAD, is associated with low-grade systemic inflammation (termed “inflammaging”) [20, 21]. Senescent cells acquire the senescence-associated secretory phenotype (SASP), characterized by increased secretion of pro-inflammatory cytokines, including IL-1β, IL-6, IL-8, chemokines, growth factors and several other freely diffusible molecules [22]. Levels of these pro-inflammatory cytokines (including IL-1β, IL-6, IL-8, IL-18 and TNF) are upregulated in serum, cerebrospinal fluid (CSF) and post-mortem brain tissue of AD patients [23–25]. Thus, aging is associated with a basal inflammatory state, which is exacerbated in AD.

AD is characterized by a state of sustained glial activation and neuroinflammation. Prolonged immune activation results in part from ineffective clearing of pathological aggregates such as Aβ. This immune response in turn activates glia and induces the secretion of pro-inflammatory cytokines, which can drive further aggregation of Aβ by affecting the capacity of microglia and astrocytes for phagocytosis and degradation [26]. Microglia migrate to sites of Aβ-rich plaques, surrounding them and phagocytosing Aβ that is dynamically released from the plaques. In line with this, increased numbers of microglia can be detected near Aβ plaques in AD. These disease-associated microglia (DAMs), which express plaque-induced genes (PIGs) in the context of AD, are activated, inflammatory and display increased phagocytic activity to clear Aβ and limit plaque-associated neurotoxicity [27]. Transcriptionally, the DAM signature includes downregulation of genes expressed in homeostatic microglia such as *CX3CR1* and *P2RY12* and upregulation of genes including *APOE, AXL, SPP1* and *TREM2*, as well as major histocompatibility complex class II (*MHC-II*), a gene essential for the initiation of antigen-specific immune response [27–29].

Several AD risk genes expressed primarily in microglia and other myeloid cells have important roles in immune function and inflammatory response both inside and outside of the brain. One such gene is triggering receptor expressed on myeloid cells 2 (*TREM2*). TREM2 is a transmembrane receptor with an immunoglobin-like ectodomain that associates with the adaptor protein DNAX-activating protein of 12 kDa (DAP12, also known as TYRO protein kinase-binding protein or TYROBP) to transduce downstream signaling. TREM2 ligands include zwitterionic and anionic phospholipids, glycolipids, apoptotic cells, lipoproteins such as HDL, LDL, APOE and APOJ/CLU, as well as Aβ [30, 31]. Autosomal recessive loss-of-function mutations in *TREM2* cause Nasu-Hakola disease, which is a rare genetic disorder characterized by bone cysts and progressive presenile dementia [32]. Coding variants in *TREM2* confer a high risk for developing AD. The most common coding variant, R47H, results in a reduction of TREM2 function and was found to increase risk for developing AD three- to five-fold, suggesting that TREM2 is protective in AD. Several other TREM2 variants associated with AD and frontotemporal dementia (FTD) have also been found to confer partial loss-of-function, as they impair TREM2 binding to ligands, maturation, ectodomain shedding and microglial phagocytic activity [33]. Microglia with *TREM2* loss-of-function cannot transition to the DAM state and are locked in a homeostatic state, in which they no longer respond normally to pathological challenges [34]. Microglia with TREM2 reduction do not cluster and become activated around plaques, which was shown to result in elevated plaque burden [35]. TREM2 binding to Aβ promotes its phagocytosis by microglia, which in turn induces secretion of pro-inflammatory cytokines IL-6 and CCL3, increases microglial proliferation, migration and apoptosis and reduces intracellular K^+^ concentrations [31, 36]. While the role of TREM2 activity in AD pathogenesis appears complex, some studies have suggested that TREM2’s exact role may depend on the context and stage of the disease. Loss of *Trem2* in an APP/PS1 AD mouse model accelerated amyloidogenesis in early disease pathogenesis, whereas *Trem2* deficiency did not affect amyloid load at a later disease stage [37]. On the other hand, another study reported reduced plaque numbers early, but increased plaque area later in disease progression in APP/PS1/*Trem2*^*-/-*^ mice [38]. In line with this, prolonged TREM2 activation by inhibiting its proteolytic cleavage in another AD mouse model increased plaque deposition and promoted neuroinflammation at an early stage of amyloidogenesis [39]. Moreover, loss of TREM2 function has been found to increase tau pathology, with elevation in tau seeding around Aβ plaques in APP/PS1 mice injected with tau isolated from human AD brain [40]. On the other hand, in PS19 tau transgenic mice that express human tau containing the P301S mutation (in the absence of plaque pathology), loss of TREM2 was protective against brain atrophy and synapse loss [41, 42], and this protective effect was abolished in the presence of ApoE4 [43]. Together, these findings point to a dynamic and complex role for TREM2 during different stages and contexts of AD pathogenesis, possibly mediated by distinct signaling pathways.

Phospholipase-Cγ2 (*PLCG2*) is another gene associated with AD risk which has a role in microglial inflammation. Mutations in the gene cause the immune disorder PLCG2-associated antibody deficiency and immune dysregulation (PLAID) and its autoimmune version APLAID, which result in immunodeficiency [44]. PLCγ2 is a membrane-associated enzyme that cleaves phosphatidylinositol-4,5-bisphosphate (PIP_2_) to generate diacyl-glycerol (DAG) and inositol-1,4,5-trisphosphate (IP_3_), which play central roles in intracellular signal transduction. While missense hypermorphic variants of *PLCG2* confer a reduced risk for AD, the mechanistic role that PLCγ2 plays in AD pathogenesis is unclear [44–48]. In human iPSC-derived microglia, PLCγ2 was shown to act downstream of TREM2 to modulate cell survival, phagocytosis, lipid metabolism and clearance of neuronal debris, while also acting downstream of toll-like receptors (TLRs) to mediate pro-inflammatory response upon microglia stimulation with lipopolysaccharide (LPS) [48]. *PLCG2* RNA expression was found to be increased in post-mortem brain tissue of LOAD patients and was positively correlated with increased amyloid burden [47]. These results suggest that like TREM2, PLCγ2 activity could be beneficial or harmful depending on the disease stage.

*INPP5D* is a gene specifically expressed in myeloid cells that encodes the protein src homology 2 (SH2) domain containing inositol polyphosphate 5-phosphatase 1 (SHIP1). While *INPP5D* has been repeatedly implicated in AD risk, it had first been recognized for being essential for proper hematopoiesis [49]. Loss-of-function mutations in *INPP5D* have been linked to myeloproliferative cancers such as acute myeloid leukemia [50–52]. Germline ubiquitous knockout of this gene in mice leads to the overproliferation of peripheral myeloid cells that results in early postnatal lethality [53]. Together, these studies point to the importance of SHIP1 function in myeloid cells. Evidence of potential dysfunction of *INPP5D* in AD will be discussed in later sections.

Together, the roles of several AD risk genes in peripheral immune disorders and inflammatory microglia activation validate the importance of inflammation and the innate immune system in AD pathogenesis. Recently, a role for inflammasomes has been recognized for mediating neuroinflammation in neurodegenerative diseases including AD. Dysregulated inflammasome activation had been previously linked to autoimmune diseases and cancer. Inflammasomes are a part of the innate immune system that coordinate host defense response against pathogens and host-derived “danger” signals that communicate cellular stress or toxicity [19, 54, 55]. The next section provides an overview of the inflammasome and its involvement in inflammation.

## The inflammasome is a multiprotein complex involved in activation of inflammatory responses

**Fig. 1:**
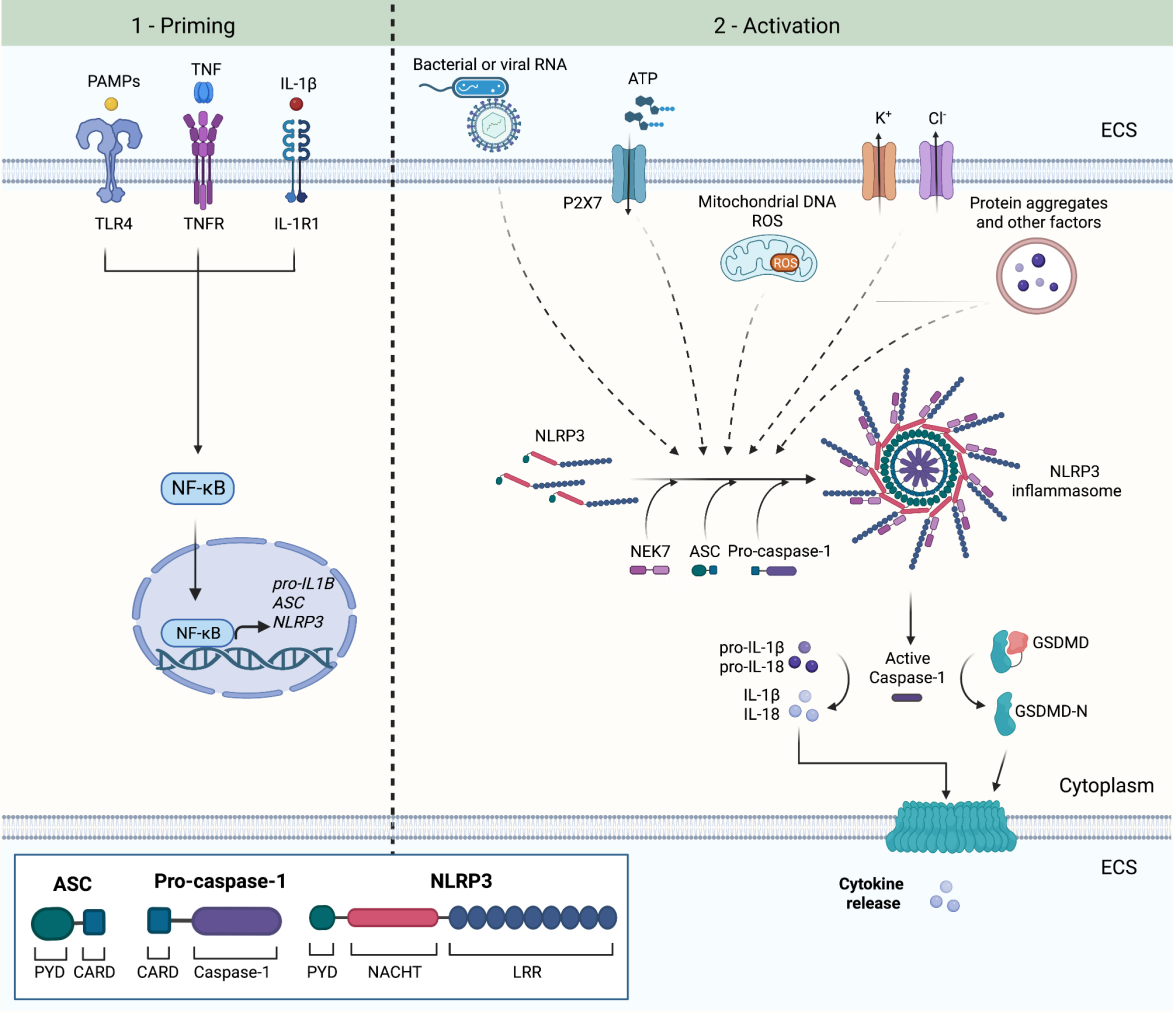
NLRP3 inflammasome activation involves two steps. In the priming step, activation of TLR4, TNFR or IL-1R1 leads to activation of NF-kB, which subsequently translocates to the nucleus and induces the transcription of *pro-IL-1β*, *ASC* (also known as *PYCARD*) and *NLRP3*. NLRP3 is autoinihibted in the cytosol and remains in the autoinhibited state after the priming step. An activating signal is necessary to release the autoinhibition. Activated NLRP3 can undergo oligomerization through homotypic NACHT domain interactions to form the inflammasome complex. NLRP3 oligomers induce the filament assembly of ASC (PYCARD) into ASC specks through homotypic PYD interactions. ASC specks act as a molecular platform to recruit pro-caspase-1 through homotypic CARD interactions. Pro-caspase-1 undergoes autoproteolysis to generate active caspase-1, which can cleave pro-forms of IL-1β and IL-18 into their mature forms. Caspase-1 can also cleave gasdermin D (GSDMD) into its N-terminal fragment (GSDMD-N), which translocates to the plasma membrane and forms a pore, through which IL-1β and IL-18 are released into the extracellular space (ECS). NLRP3, nucleotide-binding domain (NOD)-like receptor protein 3; ASC, apoptosis-associated speck-like protein; PYD, pyrin-like domain; CARD, caspase recruitment domain; LRR, leucine-rich repeat. Figure created in BioRender.com [55, 61, 62]

The canonical inflammasome complex is composed of a sensor, an adaptor, and a downstream effector. The cytosolic sensor can be either of the nucleotide-binding domain and leucine-rich-repeat-containing (NLR), absent in melanoma 2 (AIM2) or pyrin proteins. These proteins assemble the formation of a multiprotein complex, called the inflammasome, upon sensing molecular signals associated with pathogens, also called pathogen-associated molecular patterns (PAMPs), or endogenous damage/danger-associated molecular patterns (DAMPs). NLRP3 is a member of the NLR protein family that can recognize a variety of DAMPs, PAMPs, as well as environmental substances. NLRP3 inflammasomes are currently the best characterized inflammasomes and are predominantly expressed by microglia in the CNS [55]. NLRP3 protein consists of a C-terminal leucine-rich repeat (LRR), followed by an NLP family apoptosis inhibitor protein (NAIP) nucleotide-binding domain (NACHT) and an N-terminal pyrin domain (PYD) (Fig. 1). The LRR domain recognizes activating signals, the NACHT domain mediates the self-oligomerization and activation of NLRP3, while PYD mediates the downstream protein-protein interactions. The adaptor protein, apoptosis-associated speck-like protein containing a CARD (ASC, also known as PYCARD) consists of PYD and a caspase recruitment domain (CARD). Finally, the main effector caspase, caspase-1, initiates the secretion of the pro-inflammatory cytokines IL-1β and IL-18 [19, 54, 56].

NLRP3 inflammasome activation is a two-step process. The first step, often referred to as the priming step, prepares the inflammasome for activation through a series of transcriptional and post-translational mechanisms that allow the generation of components of the inflammasome. This priming step can be induced in a variety of ways including activation of TLRs upon ligand binding, which includes bacterial LPS and other PAMPs, as well as the activation of tumor necrosis factor receptor (TNFR) and IL-1 receptor 1 (IL-1R1) by exogenous TNF and IL-1β, respectively. The priming step involves upregulation of expression of inflammasome components, including *NLRP3*, *ASC*, as well as *pro-IL-1β*, all of which are upregulated through NF-kB signaling to levels necessary for inflammasome activation. In the second, activation step, the now primed NLRP3 inflammasome can be directly activated by any of the various signals including ATP, reactive oxygen species (ROS) and DNA released from damaged mitochondria, protein aggregates, uric acid crystals and ceramide, bacterial or viral RNA, airborne pollutants such as asbestos and silica, decreased intracellular concentrations of K^+^ and Cl^−^ as well as Ca^2+^ flux [57–60] (Fig. 1).

The inflammasome is tightly regulated and autoinhibited at the resting state. In the priming step, the inflammasome remains in the autoinhibited state while becoming sensitized to activating signals. Once the inflammasome components are activated, NLRP3 undergoes oligomerization through homotypic interactions between NACHT domains, which leads to the loss of the autoinhibited state. The serine-threonine kinase NIMA-related kinase 7 (NEK7) interacts and forms a complex with NLRP3, which is essential for NLRP3 activation. The oligomerized NLRP3 recruits and induces the filament assembly of ASC through homotypic PYD interactions. ASC acts as a molecular platform to recruit pro-caspase-1 through homotypic CARD interactions. Pro-caspase-1 then undergoes proximity-induced autoproteolysis, which leads to active caspase-1 processing pro-IL-1β and pro-IL-18 into their mature forms IL-1β and IL-18 and the subsequent secretion of these cytokines [55, 58, 61–63] (Fig. 1).

Inflammasome activation can lead to a form of cell death called pyroptosis, although sublethal inflammasome activation can also occur [64, 65]. During sublethal inflammasome activation, activated caspase-1 cleaves gasdermin D (GSDMD) to generate N-terminal GSDMD fragment (GSDMD-N), which inserts into and forms pores in the plasma membrane, resulting in the release of inflammatory cytokines into the extracellular environment [64, 66] (Fig. 1). While the N-terminal domains of other members of the gasdermin family (GSDMA, GSDMB, GSDMC and GSDME) can also form pores and induce necrotic cell death, GSDMD is the only gasdermin protein with a cleavage site for caspase-1 [67]. Pyroptosis may occur downstream of GSDMD pore formation and is mediated by Ninjurin1 (NINJ1), a cell surface protein that induces plasma membrane rupture upon its oligomerization [68, 69]. Cell membrane rupture upon pyroptosis leads to the release of DAMPs such as HMGB1 as well as ASC specks into the extracellular space [69, 70]. These ASC specks released from the pyroptotic cell accumulate in the extracellular space, where they can potentially continue to process and activate pro-caspase-1 and pro-IL-1β [70]. They can also be recognized by neighboring microglia as a danger signal and induce inflammatory responses including activation of the NLRP3 inflammasome, thereby propagating inflammation [70–72].

## NLRP3 inflammasome activation in microglia is implicated in AD

NLRP3 inflammasome activation is implicated in the systemic low-grade inflammation in the brain and periphery during normal aging. Various DAMPs that accumulate throughout an organism’s lifespan activate the NLRP3 inflammasome, which leads to elevated IL-1β levels associated with aging [73]. Interestingly, ablation of *Nlrp3* in mice reduced circulating levels of IL-18 and IL-1β and reduced microglial activation and astrogliosis in the hippocampus. *Nlrp3* ablation also increased expression of genes related to learning and memory and reduced age-related cognitive decline in “wild type” mice [73]. Elevated levels of IL-1β in the brain can itself induce neuroinflammation, neurotoxicity and learning and memory impairment [55, 74]. Therefore, increased baseline activation of the NLRP3 inflammasome in aging may predispose the brain to neurodegenerative diseases, in which neuroinflammation is a common feature [55].

Concordantly, aberrant NLRP3 inflammasome activation has been found to mediate AD-related neuroinflammation and exacerbate pathogenesis. Evidence for activation of functional NLRP3 inflammasomes has been detected in early and intermediate stages of AD from studies of post-mortem brains. On the other hand, only an increase in NLRP3 levels, but not in ASC or caspase-1, was observed in monocytes isolated from mildly cognitively impaired (MCI) individuals. In these monocytes, NLRP3 and caspase-1 were also not co-localized and IL-18/IL-1β secretion was not detected, suggesting that the NLRP3 inflammasome might not be active at this stage of disease [75, 76]. However, another study reported strongly increased levels of active caspase-1 in early-onset AD and LOAD patients as well as in MCI individuals [77]. These findings suggest that elevated NLRP3 inflammasome activation is associated with AD neuropathology. Indeed, distinct forms of Aβ act as DAMPs that can activate the inflammasome. Fibrillar Aβ (fAβ), following phagocytosis by microglia, activates the NLRP3 inflammasome and leads to increases in secretion of IL-1β and IL-18. fAβ phagocytosis results in lysosomal dysfunction and release of lysosomal contents into the cytoplasm, including the lysosomal protease cathepsin B, which is a mediator of inflammasome activation induced by Aβ [78]. Oligomeric Aβ (oAβ) also can activate the NLRP3 inflammasome and induce IL-1β secretion in LPS-primed microglia, but this effect was not dependent on oAβ phagocytosis and release of cathepsin B from damaged lysosomes. Instead, it was suggested that oAβ might activate the inflammasome by inducing ROS generation by damaged mitochondria and NADPH oxidase 2 (Nox2), formation of pores in the cell membrane, disruption of K^+^ channels and subsequent K^+^ efflux [79].

While Aβ can activate the NLRP3 inflammasome, it has been suggested that NLRP3 inflammasome activation also can promote Aβ aggregation. As previously mentioned, ASC specks are released into the extracellular milieu after pyroptosis, where they may propagate inflammation. In one study, ASC specks in the extracellular space could bind Aβ which then led to further Aβ aggregation both in vivo and in vitro. In that study, ASC-bound Aβ was detected in brain samples from AD patients, but not in samples from age-matched controls without dementia. Co-localization of ASC and Aβ also was found in brain tissue from patients with MCI that preceded AD, indicating that ASC speck-induced Aβ aggregation may occur in the brain [80]. ASC-binding to Aβ induced pyroptosis and impaired microglial uptake and degradation of Aβ in primary mouse microglia, contributing to further pathology driven by these aggregates [72].

The NLRP3 inflammasome has a bidirectional relationship with AD-related tau pathology as well. Evidence suggests that while some forms of tau can activate the NLRP3 inflammasome, inflammasome activation can exacerbate tau pathology by promoting elevated phosphorylation of tau [81, 82]. Treatment of wild-type primary microglia with recombinant wild-type tau and recombinant tau with a P301S mutation increased IL-1β secretion, an effect that was attenuated in *Nlrp3*^−/−^ and *Asc*^−/−^ microglia, suggesting that tau induces IL-1β secretion in a NLRP3-dependent manner [81]. On the other hand, treatment of mouse primary hippocampal neurons with conditioned media from wild-type, but not *Nlrp3*^*−*/−^ and *Asc*^−/−^, microglia primed with LPS resulted in elevated total and phosphorylated tau levels, suggesting that NLRP3 inflammasome activation in microglia can induce tau pathology in neurons. Moreover, treatment with brain homogenates from APP/PS1 mice induced tau hyperphosphorylation in a mouse model of tau aggregation pathology (Tau22) but not in Tau22/*Nlrp3*^−/−^ and Tau22/*Asc*^−/−^ mice, indicating the NLRP3 inflammasome as a mediator of Aβ-induced tau pathology [81]. In line with these findings, another study showed that aggregated tau seeds activated the NLRP3 inflammasome in primary mouse microglia by inducing lysosomal destabilization and release of the lysosomal protease cathepsin B, similar to fAβ [78, 82]. In the same study, exogenously seeded tau induced tau pathology in Tau P301S transgenic mice, but not in *Asc*-deficient or NLRP3 inhibitor (MCC950)-treated Tau transgenic mice, demonstrating the ability of the NLRP3 inflammasome to aggravate tau pathology [82].

Inhibiting or downregulating the NLRP3 inflammasome ameliorates AD neuropathology in animal models, which opens up a therapeutic avenue for modulators of NLRP3 activation in AD. Ablation of *Nlrp3* or *Casp1* in APP/PS1 mice rescued spatial memory and behavioral deficits, LTP suppression and spine loss observed in these mice [77]. APP/PS1/*Nlrp3*^−/−^ and APP/PS1/*Casp1*^−/−^ mice also displayed decreased Aβ plaque deposition, suggesting that NLRP3 inflammasome activation impairs microglial capacity to clear Aβ [77]. The decreased plaque deposition was also related to increased levels of the proteolytic enzyme insulin-degrading enzyme (IDE) [77], previously reported to degrade Aβ [83]. In Tau22 mice, ablation of *Nlrp3* or *Asc* reduced levels of hyperphosphorylated tau and tau aggregates and rescued tau-induced spatial memory deficits [81]. In light of the potential of NLRP3 ablation in alleviating AD pathology and related phenotypes, small molecule inhibitors of NLRP3 are currently being investigated as a therapeutic strategy [84]. For example, the NLRP3-specific small molecule inhibitor MCC950 was found to reduce microglial activation, IL-1β secretion and Aβ plaque numbers, increase microglial phagocytosis of Aβ and improve cognitive function in APP/PS1 mice [85, 86]. Whether these inhibitors will prove clinically useful remains to be investigated.

## SHIP1 regulates microglia function and activation

### Phosphoinositide signaling in AD

Phosphotidylinositol phosphates (PIPs), generated by the phosphorylation of phosphoinositides, are important second messengers that regulate various cellular functions in eukaryotes. In the brain, PIPs have known functions in actin remodeling, synaptic transmission, membrane trafficking, autophagy, endocytic trafficking and fusion, to name just a few. In microglia, PIPs mediate functions including phagocytosis, chemotaxis and activation.

**Fig. 2:**
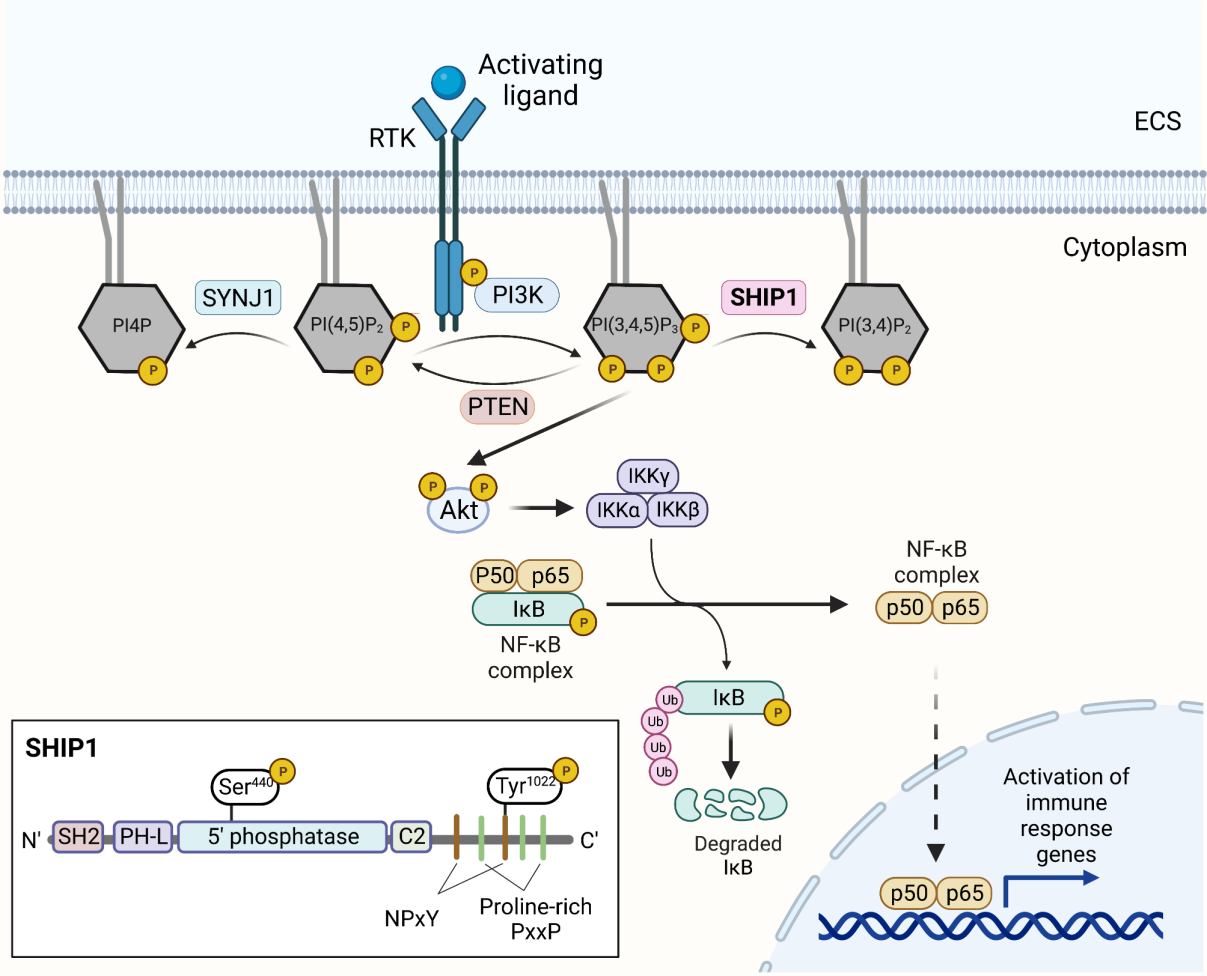
SHIP1 is a phosphatase involved in phosphoinositide signaling. Activation of a receptor tyrosine kinase (RTK) recruits the p85 subunit of phosphotidylinositol-3 kinase (PI3K). PI3K generates PI(3,4,5)P_3_ at the plasma membrane by phosphorylating PI(4,5)P_2_. SYNJ1 can dephosphorylate PI(4,5)P_2_ back into its precursor, PI4P. PI(3,4,5)P_3_ can activate Akt, which can activate various downstream targets, one of which is IKKα of the IKK kinase complex. IKKα activation leads to phosphorylation of IkB, followed by the ubiquitin-mediated proteasomal degradation of IkB, which enables the NF-kB complex to translocate to the nucleus. The NF-kB complex can activate the transcription of its numerous target genes, including genes involved in immune response. SHIP1 can negatively regulate PI3K/Akt signaling. SHIP1 binds to PI(3,4,5)P_3_ via its PH-L domain and dephosphorylates PI(3,4,5)P_3_ via its 5’ phosphatase catalytic domain to generate PI(4,5)P_2_. C2 domain of SHIP1 can bind to PI(4,5)P_2_, which is thought to allosterically regulate SHIP1’s phosphatase activity. SH2 domain can dock onto phosphorylated tyrosine residues to regulate the activity of receptors containing immunoreceptor tyrosine activating/inhibiting motif (ITAMs/ITIMs). This domain is also important for SHIP1’s localization within the cell. SHIP1 can be phosphorylated at Ser440 within the 5’ phosphatase domain by PKA to increase the phosphatase activity. Phosphorylation of SHIP1 at Tyr1022 within the NPxY motif allows binding with SHIP1’s own SH2 domain, which may mediate its dimerization or oligomerization. ECS, extracellular space; SH2, src homology 2, PH-L, plectrin homology-like. Figure created in BioRender.com [87, 88, 91, 106, 107, 128]

An important event in PIP signaling is the recruitment of phosphatidylinositol-3 kinase (PI3K) by receptor tyrosine kinases (RTKs), either directly or through adaptor proteins, which leads to the activation of PI3K. PI3K then phosphorylates PI(4,5)P_2_ to generate PI(3,4,5)P_3_, which activates Akt (also known as phosphokinase B or PKB). Activation of Akt leads to phosphorylation of numerous target proteins that in turn affect gene transcription, one of which is NF-kB, an important mediator of inflammation [87]. Activated Akt stimulates IKKα of the IKK kinase complex, which leads to the degradation of the NF-kB complex inhibitory protein IkB. Activated NF-kB can then translocate to the nucleus, where it stimulates the transcription of its target genes, some of which include immune response genes and inflammasome components [88]. PI(3,4,5)P_3_ can be converted back to PI(4,5)P_2_ by the protein phosphatase and tensin homology (PTEN) or converted into PI(3,4)P_2_ by SHIP1, which negatively regulates PI3K/Akt signaling (Fig. 2) [89–92].

Altered PI(4,5)P_2_ metabolism has been linked to Aβ toxicity [93–95]. Familial AD *PSEN1* mutations result in disrupted metabolism of PI(4,5)P_2_ [95] and PI(4,5)P_2_ has been reported to be reduced in the AD brain [93]. Further evidence of the importance of phosphoinositide signaling in neurodegenerative disease comes from studies of SYNJ1, the primary PI(4,5)P_2_ phosphatase. *SYNJ1* is on chromosome 21 and is elevated in Down syndrome (DS) brains. DS mouse models show disrupted PI(4,5)P_2_ metabolism that is caused by an extra copy of *Synj1* [96, 97]. Evidence that PI(4,5)P_2_ metabolism mediates the effects of Aβ oligomers on synaptic health comes from in vitro experiments. Treatment of primary cortical neurons with oligomeric Aβ (but not monomeric or fAβ) induces a rapid reduction in PI(4,5)P_2_ levels that is reversible with removal of the treated Aβ [94]. This effect of Aβ oligomers was attenuated in primary neurons from mice with loss of one copy of *Synj1* [94].

### SHIP1 modulates downstream activity of multiple receptors involved in microglia activation and function

SHIP1 was originally observed in hematopoietic cells after a variety of cytokines and growth factors induced the tyrosine phosphorylation of this 145-kDa protein [98–104]. In the brain, expression of SHIP1 is primarily restricted to microglia [105]. SHIP1 consists of a Src homology 2 (SH2) domain near its N-terminus, which docks onto phosphorylated tyrosine residues, followed by a plectrin homology-like (PH-L) domain that binds to PI(3,4,5)P_3_ and localizes SHIP1 to the membrane [106]. Upon binding to PI(3,4,5)P_3_, the 5’ phosphatase catalytic domain phosphorylates PI(3,4,5)P_3_ to generate PI(3,4)P_2_, which can bind to the C2 domain near the C-terminus [92, 107, 108]. The C-terminus of SHIP1 contains a NPxY motif, which can bind to proteins containing immunoreceptor tyrosine inhibitory motifs (ITIM)/immunoreceptor tyrosine activating motifs (ITAM), phosphotyrosine-binding (PTB) or SH2 domains, as well as a proline-rich PxxP motif that contains SH3-binding domains [47, 93–98] (Fig. 2). The 5’ phosphatase domain and the NPxY motif can be post-translationally modified: protein kinase A (PKA) phosphorylation at Ser^440^ of the 5’ phosphatase domain increases the phosphatase activity of SHIP1 [109, 110], while phosphorylation at Tyr^1022^ (Tyr^1020^ in mice) within the NPxY motif allows SHIP1 to bind PTB domain-containing proteins [111, 112], as well as SHIP1’s own SH2 domain [113]. The binding of the SH2 domain and Tyr^1022^ has been postulated to mediate SHIP1’s possible dimerization or oligomerization and sequester the SH2 domain from binding to ITIMs/ITAMs [113].

**Fig. 3:**
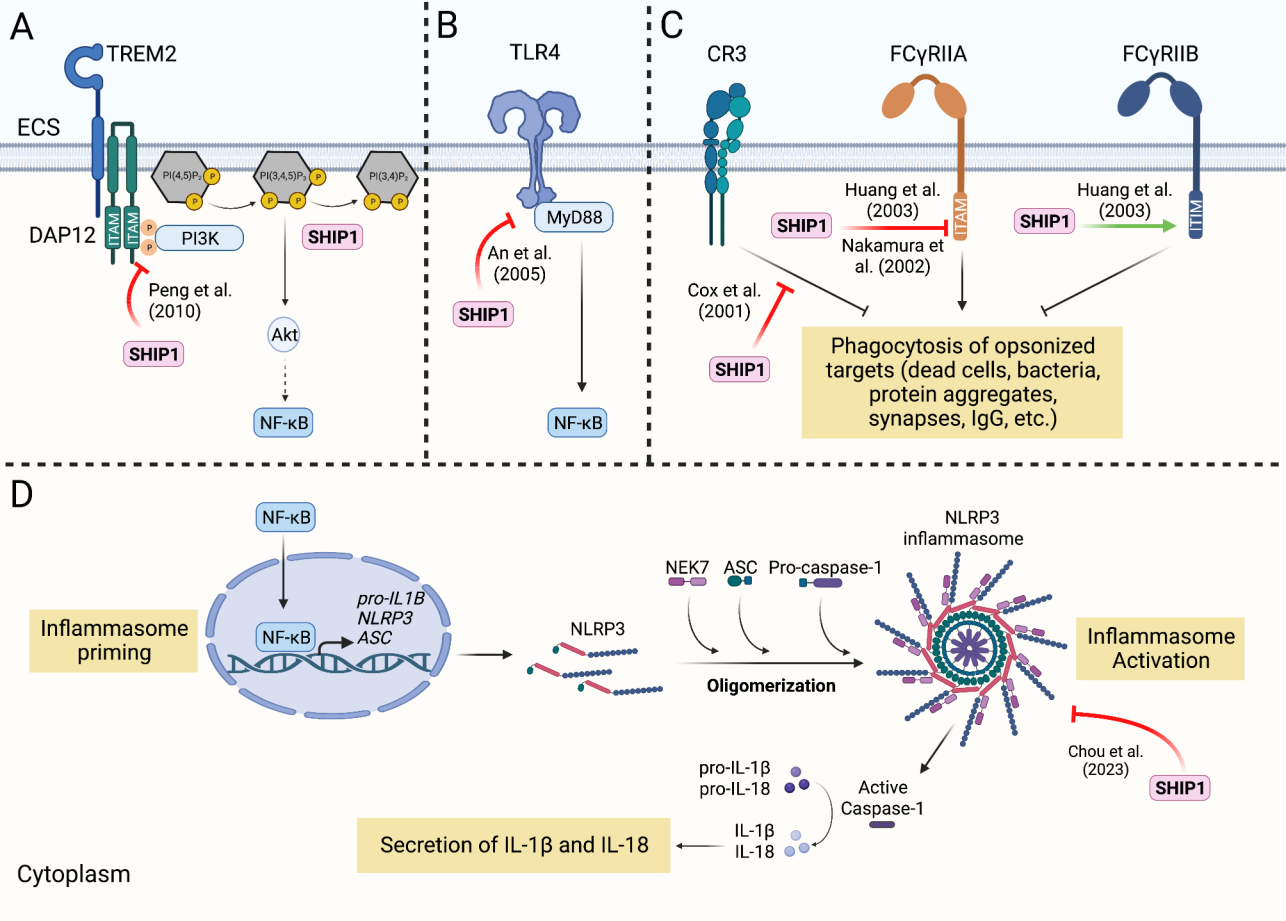
SHIP1 regulates multiple receptors expressed in microglia and the microglial NLRP3 inflammasome. (**A**) TREM2 activation recruits p85 subunit of PI3K to the immunoreceptor tyrosine activating motif (ITAM) of DAP12. SHIP1 inhibits downstream activity of TREM2/DAP12 by binding to the immunoreceptor tyrosine activating motif (ITAM) of DAP12, preventing the association of p85 with DAP12, in osteoclasts. (**B**) SHIP1 inhibits the association of TLR4 with its adaptor protein MyD88 in macrophages. TLR4/MyD88 signaling leads to activation of NF-kB by inducing degradation of IΚB, which is also inhibited by SHIP1. (**C**) SHIP1 inhibits phagocytosis mediated by CR3 and FCγRIIA in macrophages. SHIP1 binds to the ITAM of FCγRIIA, inhibiting the downstream PI3K signaling. SHIP1 can also bind to the immunoreceptor tyrosine inhibitory motif (ITIM) of the inhibitory receptor FCγRIIB, which enhances FCγRIIB-mediated inhibition of phagocytosis. The exact mechanism of how SHIP1 inhibits CR3 is currently unknown. (**D**) SHIP1 inhibits the activation of the NLRP3 inflammasome. Pharmacological inhibition of SHIP1 or genetic reduction of *INPP5D* activates the NLRP3 inflammasome in human iPSC-derived microglial cells (iMGs), resulting in increased secretion of IL-1β and IL-18. On the other hand, loss of SHIP1 inhibits the upregulation of *pro-IL-1β*, suggesting that reduction of SHIP1 activity may induce inflammasome activation while inhibiting the priming step in some contexts. ECS, extracellular space. Figure created in BioRender.com [114, 121, 137, 138, 161, 197]

SHIP1 can modulate microglia function and activation by modulating distinct but converging signaling pathways. SHIP1 has been proposed to inhibit TREM2 signaling by binding to the ITAM of DAP12 in osteoclasts. This prevents the association of the p85 regulatory subunit of PI3K with the ITAM of DAP12 through its own SH2 domain, suggesting that SHIP1 inhibits PI3K-mediated signaling events downstream of TREM2/DAP12 [114] (Fig. 3A). Ongoing efforts are underway to clarify the mechanisms by which SHIP1 signaling may interact with TREM2 signaling to mediate processes relevant to AD.

Despite evidence that TREM2 can activate PI3K/AKT signaling, TREM2 activation has been largely associated with an anti-inflammatory role. Some studies have suggested that the anti-inflammatory effect of TREM2 is mediated by its inhibition of PI3K/Akt signaling and subsequent NF-kB activation [114–117]. This seemingly contradictory relationship between TREM2 and PI3K/AKT signaling was mechanistically consolidated to some extent by another study, which proposed that TREM2-dependent phagocytosis is mediated by TREM2’s activation of PI3K/AKT signaling, whereas TREM2’s suppression of NF-kB is independent of PI3K/Akt but might instead be dependent on PKC [118]. On the other hand, TREM2 can be directly bound and activated by Aβ, after which it mediates Aβ phagocytosis as well as Aβ-dependent microglial activation, cytokine production and secretion as previously mentioned [31]. While TREM2 function has been classically considered anti-inflammatory and protective in AD, its interaction with Aβ can trigger Aβ-mediated inflammation and exacerbate Aβ deposition, ultimately linking TREM2 with a pro-inflammatory and even a detrimental role depending on the context and disease stage [31, 36]. For an extensive review of TREM2 functions and its role in neurodegenerative disease, readers are encouraged to refer to Shi and Holtzman (2018) [119], Lewcock et al. (2020) [120] and Hou et al. (2022) [30].

SHIP1 also has been shown to modulate toll-like receptor 4 (TLR4) signaling, associated with microglial activation and inflammation upon recognizing PAMPs and DAMPs, in mouse bone marrow-derived dendritic cells and macrophages [121]. TLR4, like other members of the TLR family, is a pattern recognition receptor that recognizes exogenous molecular patterns belonging to pathogens, such as bacterial LPS, or endogenous molecular patterns, such as protein aggregates [122]. Within plaque-associated microglia, Aβ fibrils upregulate microglial cytokine, chemokine and ROS production via TLR4 signaling [123, 124]. TLR4 as well as several other TLRs are implicated in the clearance of Aβ deposits [125]. RNA expression of *TLR4* is increased in APP/PS1 mice and in brain tissue of patients with AD in association with plaque deposition [126, 127]. Activation of TLR4 results in the tyrosine phosphorylation of its cytosolic adaptor protein myeloid differentiation primary response 88 (MyD88), which can recruit the p85 subunit of PI3K and activate PI3K/Akt signaling [116]. In mouse macrophages, the overexpression of SHIP1 was found to inhibit the LPS-induced TLR4 signaling and the subsequent production of the pro-inflammatory cytokines TNF-α and IL-6 by negatively regulating the interaction between TLR4 and MyD88, though whether this regulation is dependent on SHIP1’s phosphatase activity remains unclear [121, 128] (Fig. 3B).

SHIP1 has been recognized as a negative regulator of inflammation mediated by various signaling pathways in macrophages. Besides TLR4, another pattern recognition receptor whose activity is regulated by SHIP1 is NOD2, a cytosolic bacterial sensor and a member of the NLR family of pattern recognition receptors. Stimulation of NOD2 upon bacterial invasion activates NF-kB signaling and may lead to caspase-1-dependent IL-1β secretion, similar to the events downstream of NLRP3 inflammasome activation [129]. SHIP1 was found to inhibit NF-kB activation and the subsequent induction of *IL-6, IL-8* and *TNF-α* expression in human monocytes and mouse bone marrow-derived macrophages independent of its phosphatase activity [130]. While SHIP1 downregulates pro-inflammatory cytokine production, it also can suppress inflammation by supporting anti-inflammatory cytokine activity [131]. In macrophages, the anti-inflammatory cytokine IL-10 can suppress TNF-α secretion, which is promoted by both phosphatase-dependent and -independent activities of SHIP1 [131–133]. SHIP1’s role in inflammation in microglia, the resident macrophages of the CNS, will be discussed in the future sections.

Multiple studies support the hypothesis that SHIP1 negatively regulates microglia and macrophage phagocytosis, likely through its proposed regulation of TREM2 and TLR4, as well as Fc gamma receptor (FCγR) and complement receptor 3 (CR3)-mediated signaling, and potentially of other signaling pathways [106, 114, 121, 134–138]. FCγRs bind to the constant domain of immunoglobins (IgG) and are expressed on the surface of immune cells, including microglia. Activation of FCγRs leads to a pro-inflammatory response, marked by secretion of cytokines and chemokines. FCγR activation also promotes Aβ clearance via phagocytosis [139, 140]. In mouse macrophages and COS cells, SHIP1 was shown to activate the ITIM of the inhibitory receptor FCγRIIβ, which inhibits the activating receptor FCγRIIA to block FCγRIIA-mediated phagocytosis (Fig. 3C**).** SHIP1 also was shown to directly inhibit FCγRIIA by binding to its ITAM (Fig. 3C), which prevents the activation of PI3K [134, 137–140]. Therefore, SHIP1 inhibition of FCγRIIA-mediated signaling shares a similar mechanism with SHIP1’s inhibition of TREM2 signaling, both of which ultimately downregulate phagocytosis.

SHIP1 also has been shown to modulate complement receptor 3 (CR3)-mediated phagocytosis in macrophages [134, 138, 141]. CR3 (also known as CD11b/CD18 or Mac-1) is a major phagocytic receptor in the complement system, a large family of proteins that are activated in a cascade-like manner in response to pathogens by the innate immune system. CR3, along with other complement proteins, mediate microglial synapse elimination during development [15, 142]. In AD, CR3-mediated elimination of synapses was proposed to lead to the aberrant synapse loss seen early in the disease progression [142, 143]. Although CR3 mediates the clearance of fibrillar and oligomeric Aβ, it might also promote the accumulation of Aβ deposits [144, 145]. Wild-type SHIP1, but not its catalytically inactive form, was found to inhibit the phagocytosis of a ligand selective for CR3 in mouse macrophages, suggesting that the phosphatase activity of SHIP1 is required for its inhibition of CR3-mediated phagocytosis [138] (Fig. 3C). While the exact mechanism of how SHIP1 inhibits CR3-mediated phagocytosis is unknown, a possible mechanism is through SHIP1’s regulation of PI3K, whose activity is required for CR3-mediated phagocytosis [138]. Another related, potential mechanism is through SHIP1’s dephosphorylation of PI(3,4,5)P_3_ that accumulates at the phagocytic cup during CR3-mediated phagocytosis [134, 146].

In addition to its functional interactions with the receptors mentioned above, SHIP1 also may regulate phagocytosis through the TAM (named after Tyro3-Axl-Mer) receptor tyrosine kinase family. TAM receptors initiate phagocytosis upon binding of Gas6 or Pros1, two “bridging” proteins that recognize phosphotidylserine exposed on the surface of phagocytic substrates such as apoptotic cells. Activation of the tyrosine kinase of TAM receptors upon ligand binding recruits the p85 subunit of PI3K and activates PI3K/Akt signaling [147–150]. This also leads to activation of NF-kB, which is thought to promote cell survival in fibroblasts and endothelial cells [147, 151, 152]. Although TAM receptor-signaling has been classically associated with an anti-inflammatory role, TAM receptors mediate Aβ phagocytosis, which in turn promoted development of dense-core plaques [153]. In line with these observations, mRNA of *Axl* is strongly upregulated in plaque-associated microglia in AD [153–155]. Therefore, it is possible that like TREM2 signaling, interaction of TAM receptors with Aβ could propagate Aβ-mediated inflammation within microglia, resulting in a net pro-inflammatory effect [149, 153, 156–160]. Acute pharmacological inhibition of SHIP1 with 3α-aminocholestane (3AC) in human iPSC-derived microglial cells (iMGs) upregulated the mRNA and protein expression of Axl [161]. Future studies are warranted to confirm whether the increase in Axl due to SHIP1 inhibition reflect an increased activity of this receptor.

In agreement with SHIP1’s role in inhibiting phagocytosis, pharmacological inhibition of SHIP1 and knockout of *Inpp5d* in mice increased the phagocytosis of apoptotic neurons and Aβ_42_, though plaque burden still increased [135, 136]. In line with this, activation of AXL and TREM2, while promoting Aβ clearance, were found to increase plaque deposition in separate studies [36, 153]. However, this plaque deposition may be beneficial. TREM2 and TAM receptors were suggested to aid in the compaction of amyloid plaques from a diffuse form, which are more neurotoxic and promote neurite dystrophy, to a dense-core form associated with lower neurotoxicity [153, 162, 163]. Similarly, loss of one copy of *Inpp5d* in mice in the 5xFAD background increased the proportion of dense-core plaques over diffuse plaques [164]. Moreover, in AD mouse models, the number of microglia per plaque was reduced when *Trem2* or *Axl* are knocked out, whereas loss of one or both copies of *Inpp5d* increased the number of microglia that surround plaques [136, 153, 163, 164]. Expression of both *Trem2* and *Axl* are upregulated in disease-associated microglia in AD mouse models likely in response to the presence of plaques [155]. In APP/PS1 mice, knockout of *Inpp5d* further upregulated the RNA levels of *Trem2* and *Axl* in microglia that surround plaques [136]. In the cortex of 5xFAD mice, a ~ 50% reduction in *Inpp5d* increased protein levels of TREM2 and phosphorylated SYK, which indicates increased TREM2 signaling [164]. Another study generated an *Inpp5d*^+/−^;*Tyrobp*^−/−^*App* knock-in mouse model and found that *Inpp5d* haplodeficiency in addition to *Tyrobp* deficiency partially rescued the impairments in microglial response to Aβ plaques and plaque compaction observed with *Tyrobp* deficiency alone [165]. Together, these findings suggest that reduction of SHIP1 levels may increase TREM2 and AXL activity, resulting in increased phagocytosis of Aβ, microglial recruitment to plaques and plaque compaction, perhaps resulting in limiting plaque toxicity.

## Complex SHIP1 post-transcriptional regulation in AD

Given SHIP1’s potential role in regulating the signaling mechanisms discussed above, a reduction in SHIP1 protein levels is poised to promote microglial activation and inflammation related to AD pathogenesis. GWASs have identified three single nucleotide polymorphisms (SNPs), rs35349669, rs10933431, and rs7597763, at the *INPP5D* locus that are associated risk for AD [8–10]. While variant rs35349669 is proposed to confer increased risk for AD, rs61068452 is proposed to confer protection [166, 167]. These SNPs are located in introns of INPP5D, and it is unclear whether and how they affect *INPP5D* mRNA levels or splicing.

One study found elevated *INPP5D* mRNA expression in human AD brains that positively correlated with amyloid plaque density, and elevated *Inpp5d* mRNA and protein expression in 5xFAD mice throughout disease progression [168]. SHIP1 protein expression was identified in plaque-associated microglia of 5xFAD mice, with phagocytic microglia showing a reduction of *Inpp5d* expression compared to non-phagocytic microglia [168, 169]. Another study similarly found an upregulation of *INPP5D* RNA expression in both human AD patients and an *App* knock-in mouse model [170]. A recent study investigated RNA expression of the different *INPP5D* isoforms in the post-mortem brains of AD and non-AD individuals. While they also found an increase in the expression of *INPP5D* isoforms in the AD brain, the expression of a truncated isoform that lacks the phosphatase domain was increased, suggesting that the increase in *INPP5D* expression may not necessarily lead to increased SHIP1 activity [171]. Of note, the investigation of AD-associated SNPs in *INPP5D* in this study did not reveal a clear SNP effect on overall *INPP5D* expression [171].

While data from our group agrees with the elevation of *INPP5D* mRNA levels in human AD post-mortem brains, we also found that aqueous-soluble SHIP1 protein levels are reduced in brain tissue from individuals diagnosed with AD compared with non-AD individuals [161]. However, we found that AD brains displayed increased immunostaining for SHIP1 compared to age and sex-matched brain tissue from non-cognitively impaired individuals when a C-terminally directed antibody to SHIP1 was used. Plaque-associated microglia clearly displayed higher SHIP1 immunostaining than microglia distal to plaques, SHIP1 levels significantly correlated with microglial ramification, where less ramified and putatively more activated plaque-associated microglia had lower levels of SHIP1 immunostaining [161]. Moreover, the staining pattern for SHIP1 was diffuse in some microglia, while a more punctate pattern was observed in other microglia. In AD brain tissue, SHIP1 localization was shifted from diffuse to punctate patterns in microglia, suggesting that SHIP1 may be aggregated (and perhaps aqueous-insoluble) in the AD brain [161]. Peptide-level mass spectrometry analyses of TBS- and urea-solubilized postmortem brain lysates revealed a reduction of peptides encoding the phosphatase domain of SHIP1 in the AD brain [161]. Further, in agreement with a reduction in function of INPP5D in AD microglia we observe some concordance between expression profiles of iMGs with biallelic loss-of-function alleles in *INPP5D* and in microglia from AD brain tissue. For example, iMGs with biallelic *INPP5D* LOF alleles and microglia from AD brain tissue both show a reduction in RNA levels of NF-kB pathway genes. In accord, overexpression of *INPP5D* in iMGs showed an elevation in RNA levels of NF-kB pathway genes, suggesting that microglia from AD brains may display changes relevant to reduced SHIP1 activity [161]. Together, these findings support the hypothesis that there are multiple pools of SHIP1 in the brain, and that in the AD brain, enzymatically active SHIP1 is reduced. Additional analyses are warranted to develop sensitive methods to measure SHIP1 activity in microglia present in postmortem brain samples to determine if activity is altered in AD brain tissue.

In contrast to the notion that SHIP1 activity is reduced in the AD brain, recent studies in mice point to a largely beneficial role for reduction of *Inpp5d* expression. However, data regarding the impact of reduced *Inpp5d* expression on Aβ plaque load are complicated. Conditional knock out of *Inpp5d* in myeloid cells in 3-month-old APP/PS1 mice resulted in an increase in plaque burden and recruitment of microglia to plaques. In this same study, significant congruence was observed between plaque-associated gene expression changes induced by *Inpp5d* reduction and previously identified human AD gene networks [136]. However, another recent study that knocked out *Inpp5d* in myeloid cells at 6 weeks of age found reduced plaque load, accompanied by a reduction in dystrophic neurites and an increase in GFAP^+^ immunoreactivity [172]. Investigation of 5xFAD mice with germline deletion of one copy of *Inpp5d* similarly showed increased microglia engagement with plaques but decreased plaque deposition. Further, *Inpp5d* reduction rescued a behavioral phenotype that is observed in 5xFAD mice WT for *Inpp5d* at 6 months of age, reduced spontaneous alternation [164]. In another recent study, conditional knock out of *Inpp5d* in 3-week-old 5xFAD mice similarly enhanced microglia recruitment to plaques, decreased microglial ramification, and ameliorated neuritic dystrophy but had no impact on plaque burden [173]. The contradictory impacts of *Inpp5d* loss on plaque load in these studies may be attributed to the time point in disease progression at which *Inpp5d* expression is reduced as well as the differences in the AD mouse models, as also discussed recently in Gandy and Ehrlich (2023) [174].

Taken together, while these studies in humans and mice all support a role for SHIP1 in AD, there is seemingly contradictory evidence regarding whether therapeutic strategies for AD should aim to increase or decrease SHIP1 activity. While SHIP1 inhibition is currently being investigated by the field in light of the largely beneficial effects of *Inpp5d* knockdown in AD mouse models, SHIP1 agonism also has been proposed as a therapeutic strategy for AD. The differences in human and mouse studies may reflect genuine biological differences between species and/or may be due instead to differences in the disease stage interrogated and context of other, currently unknown extenuating factors. Deep analyses to disentangle these possibilities will clarify the optimal strategy for SHIP1 targeting while at the same time illuminating a better understanding regarding the fundamental biological processes at play within microglia in the healthy and diseased brain.

## Reduction of SHIP1 activity activates the NLRP3 inflammasome

Corroborating SHIP1’s role in modulating microglial activation and inflammation, we recently discovered a direct connection between SHIP1 and the NLRP3 inflammasome in iMGs. Acute pharmacological inhibition of SHIP1’s phosphatase activity as well as genetic ablation of a single copy of *INPP5D* in iMGs induced the secretion of IL-18 and IL-1β that was blocked by NLRP3 inhibition with MCC950 and also with caspase-1 inhibitors [161]. Though reduction of *INPP5D* increased the secretion of IL-18 and IL-1β, it lowered their mRNA expression, suggesting that reduction of SHIP1 activity positively regulates the activation step but may act through a feedback mechanism to inhibit the priming step for the NLRP3 inflammasome [161] (Fig. 3D).

Evidence from post-mortem analyses of human AD brains support the inverse relationship between SHIP1 levels and the activation of the inflammasome. When levels of secreted IL-1β and IL-18 were compared to aqueous-soluble SHIP1 levels in brain lysates, lower SHIP1 levels were signficantly associated with elevated IL-18 protein levels, with the correlation driven by data from AD brain tissue. Further, comparison of immunoreactivity for SHIP1 and ASC specks in brain tissue from aged individuals revealed that lower SHIP1 levels were significantly correlated with a higher percentage of microglia with ASC specks in the AD brain [161]. Furthermore, a recent single-nucleus RNA sequencing profiling study of microglia from AD and non-AD brains identified an “inflammatory” cluster that was over-represented in the AD brains. While this cluster showed significant downregulation of *INPP5D*, *IL-1β* expression was significantly elevated, and this cluster was enriched for NLR signaling [175]. Similarly, the gene expression profiles of microglia from an *App* knock-in mouse model identified an inflammatory reactive microglia cluster that was enriched with AD risk genes, including *Apoe*, but this cluster displayed decreased expression of *Inpp5d*, suggesting *Inpp5d* reduction as a potential mediator of inflammatory response in this microglia subtype [176]. Therefore, we posit that SHIP1 is a negative regulator of NLRP3 inflammasome activation and that reduction of SHIP1 activity contributes to the activation of the NLRP3 inflammasome in AD. In parallel, reduction of SHIP1 activity is poised to disinhibit signaling pathways that are involved in the phagocytosis of Aβ, which is a required step for Aβ-induced activation of the NLRP3 inflammasome [78].

Collectively, these results suggest an important role for SHIP1 in microglial biology at the non-disease state and in AD pathogenesis. SHIP1 levels might be differentially altered based on disease stage, microglia state or levels of pathological burden, and studying SHIP1 biology with these caveats in mind might reveal therapeutic avenues that can be tailored to these different contexts.

## Consequences of microglial NLRP3 inflammasome activation on other cell types in the brain

Although the inflammasome can found in many cell types in the brain, the inflammasome, and in particular the NLRP3 inflammasome, has been most commonly studied in microglia [177, 178]. While details of the inflammasome pathway have been deeply studied, much less is known about the functional consequences of inflammasome activation in immune cells on other cell types in the brain and the signaling pathways altered in these other cell types. In this section, we consider the consequences of microglial NLRP3 inflammasome activation on other brain cell types.

Microglia actively regulate neuronal synapses in a healthy physiological setting, and processes mediating this function can become aberrant in a neurodegenerative disease context, as in early stages of AD [15, 143, 179]. Microglial NLRP3 inflammasome activation can perturb neuronal morphology and function during the chronic inflammation that is observed in AD. Multiple groups have shown that inhibition of the NLRP3 inflammasome can rescue deficits in neuronal synaptic transmission and plasticity as well as reduction of neurite number and integrity that results from LPS administration in mice [180–182]. These results provide evidence that the NLRP3 inflammasome mediates microglial contributions to neurodegeneration, as inflammasome activation has deleterious effects on neuron physiology and health.

Microglial activation or inflammatory insults induce astrocytes into a reactive state, which can have both neuroprotective and neurotoxic effects [17, 183–185]. It is thought that the “neurotoxic” reactive astrocytes associated with AD release pro-inflammatory cytokines and can no longer support neuronal synapse formation, facilitate synaptic pruning and clearance of myelin debris and may even induce cell death in neighboring neurons, thereby promoting further neurodegeneration and inflammation [17, 186]. Conversely, the “neuroprotective” phenotype of astrocytes promotes neuronal survival and homeostasis, as these astrocytes secrete neurotrophic factors and anti-inflammatory cytokines [184, 187, 188]. Currently, published studies suggest that NLRP3 inflammasome activation and subsequent secretion of IL-18 and IL-1β from microglia drives astrocytes into the neurotoxic reactive state, which induces upregulation of the secretion of the complement protein C3 and results in lower synapse numbers [180, 189–191]. However, these studies evaluated astrocyte state upon NLRP3 inflammasome activation based on the binary “A1/A2” astrocyte description, which does not fully capture the diversity of astrocyte states and phenotypes in different contexts [183]. Therefore, further studies are warranted to evaluate changes in astrocyte state and function upon microglial NLRP3 activation in an unbiased manner, without, for example, testing the expression of a selected “cocktail” of marker genes.

Microglial activation of the NLRP3 inflammasome can also have deleterious effects on oligodendrocyte biology. Aberrant activation of the microglial NLRP3 inflammasome has been implicated in the onset and progression of multiple sclerosis (MS), an autoimmune demyelinating disease of the CNS [192, 193]. In a commonly employed mouse model for MS, experimental autoimmune encephalomyelitis, microglial NLRP3 inflammasome activation resulted in pyroptosis of oligodendrocytes, promoted inflammation and induced demyelination [194, 195]. These results invite speculations for a potential role of the microglial NLRP3 inflammasome activation in loss of oligodendrocytes and demyelination also seen in AD [196].

## Conclusions and perspectives

Genetic studies have revealed new insights into the importance of microglia in pathways leading to AD. These studies have inspired the development of a multitude of new rodent and human stem cell experimental systems for studying the function of these genes. The use of these systems, coupled to studies of brain tissue from AD patients, have yielded insights into aspects of the mechanisms that link dysfunction of LOAD genes to the accumulation of plaques and tangles, loss of synapse and dementia. Additionally, mechanistic studies of diseases of peripheral myeloid cells have provided clues into the signaling pathways involved in inflammasome activation and the molecular consequence of dysfunction of genes such as *INPP5D*. While these studies have yielded new insights regarding AD pathogenesis, there remain many unanswered questions that will need to be answered to translate these findings into new therapeutic strategies for AD.

Studies of the function of myeloid-expressed LOAD candidate genes have revealed that the molecular pathways involved are complex and tightly regulated. A recurring theme is that alterations of these genes have context-dependent effects that can be differential with disease stage and exposure to different environmental cues. This appears to be true for SHIP1 function and the role of phosphoinositide signaling broadly in brain health, as well as for the signaling pathways involved in priming and activation of the inflammasome. Understanding the intricate interplay between Aβ and tau clearance, phosphoinositide signaling and inflammasome activation will be important for identifying appropriate therapeutic strategies for AD. In addition, key next steps will involve better understanding the mechanisms linking these processes in microglia and other immune cells to consequences on other cell types in the brain that lead to dementia.

## Data Availability

Not applicable.

## List of abbreviations

3AC: 3α-aminocholestane
AD: Alzheimer’s disease
AIM2: Absent in melanoma 2
APOE: Apolipoprotein E
APP: Amyloid precursor protein
ASC: Apoptosis-associated speck-like protein
Aβ: Amyloid β
CARD: Caspase recruitment domain
CNS: Central nervous system
CR3: Complement receptor 3
CSF: Cerebrospinal fluid
DAM: Disease-associated microglia
DAMP: Damage/danger-associated molecular patterns
DAP12: DNAX-activating protein of 12 kDa
DS: Down syndrome
fAβ: Fibrillar amyloid β
FCγR: Fc gamma receptor
FTD: Frontotemporal dementia
GSDMD: Gasdermin D
GWAS: Genome wide association study
ICC: Immunocytochemistry
IgG: Immunoglobin
IL-18: Interleukin-18
IL-1R1: Interleukin 1 receptor 1
IL-1β: Interleukin-1β
IL-6: Interleukin-6
IL-8: Interleukin-8
iMG: Induced pluripotent stem cell-derived microglial cell
iPSC: Induced pluripotent stem cell
ITAM: Immunoreceptor tyrosine activating motifs
ITIM: Immunoreceptor tyrosine inhibitory motifs
LOAD: Late-onset Alzheimer’s disease
LOF: Loss-of-function
LPS: Lipopolysaccharide
MCI: Mildly cognitively impaired
MS: Multiple sclerosis
MyD88: Myeloid differentiation primary response 88
NAIP: NLP family apoptosis inhibitor protein
NEK7: NIMA-related kinase 7
NINJ1: Ninjurin1
NLR: Nucleotide-binding domain and leucine-rich-repeat
NLRP3: Nucleotide-binding domain (NOD)-like receptor protein 3
oAβ: Oligomeric amyloid β
PAMP: Pathogen-associated molecular patterns
PH-L: Plectrin homology-like
PI3K: Phosphatidylinositol-3 kinase
PIG: Plaque-induced genes
PIP: Phosphotidylinositol phosphate
PKA: Protein kinase A
PLCγ2: Phospholipase-Cγ2
PS1: Presenilin 1
PYD: Pyrin-like domain
ROS: Reactive oxygen species
RTK: Receptor tyrosine kinase
SASP: Senescence-associated secretory phenotype
SH2: Src homology 2
SHIP1: Src homology 2 (SH2) domain containing inositol polyphosphate 5-phosphatase 1
TAM: Tyro3-Axl-Mer
TBS: Tris-buffered saline
TLR: Toll-like receptor
TLR4: Toll-like receptor 4
TNF: Tumor necrosis factor
TNFR: Tumor necrosis factor receptor
TREM2: Triggering receptor expressed on myeloid cells 2
